# Global Disparities in Simulation-Based Learning Performance: Serial Cross-Sectional Mixed Methods Study

**DOI:** 10.2196/52332

**Published:** 2025-08-11

**Authors:** Kashish Malhotra, Harshin Balakrishnan, Emily Warmington, Vina Soran, Francesca Crowe, Dengyi Zhou, SIMBA AND CoMICs Team, Punith Kempegowda

**Affiliations:** 1 Department of Surgery, Dayanand Medical College, Punjab, India; 2 Department of Applied Health Sciences, College of Medical and Dental Sciences, University of Birmingham, Edgbaston, Birmingham, B15 2TT, United Kingdom, 44 7721930777; 3 College of Medical and Dental Sciences, University of Birmingham, Birmingham, United Kingdom; 4 Northwick Park Hospital, London North West University Healthcare NHS Trust, London, United Kingdom; 5 Queen Elizabeth Hospital, University Hospitals Birmingham NHS Foundation Trust, Birmingham, United Kingdom

**Keywords:** simulation, medical education, SIMBA, Global Rating Scale, low- and middle- income countries, high-income countries, global medical education, clinical training, gender equity, global disparity, disparity, discrepancy, inequality, health care education, clinical, cross-sectional study, linear regression model, physical examination, simulation-based learning

## Abstract

**Background:**

Simulated programs provide health care professionals (HCPs) with a learning opportunity to develop clinical competencies and improve patient outcomes in a safe and controlled environment. While the benefits of simulation training are well established, there is a paucity of research assessing its differential impact, if any. SIMBA (Simulation via Instant Messaging for Bedside Application) provides simulation-based learning through WhatsApp and Zoom (Zoom Video Communications, Inc) to increase HCPs’ confidence in managing various medical conditions.

**Objectives:**

This study aims to explore whether there are differences in the clinical performance of HCPs participating in SIMBA sessions based on gender, country of work, and training grade.

**Methods:**

This study assessed participants in 17 SIMBA sessions from May 2020 to June 2022. WhatsApp chats containing participants’ approach to the simulated scenarios were graded using an adapted version of the Global Rating Scale consisting of 6 domains: eliciting history; physical examination; investigations, diagnostic tests, and imaging; interpretation of investigations and imaging; clinical judgment; and management and follow-up or discharge plan. These domains were rated using a Likert-type scale of 1 (not done) to 5 (excellent) prior to the session based on expert inputs. All WhatsApp transcripts were evaluated against the scale postsimulation session. Unadjusted and adjusted means and 95% CIs of the scores for the 6 performance variables were calculated using multiple linear regression models. The *P* value for heterogeneity between the mean performance scores was calculated using likelihood ratio tests by using an analysis of variance.

**Results:**

A total of 289 participants across 49 countries who completed pre-SIMBA and post-SIMBA surveys in the 17 simulation sessions were included in the analysis. Participants from high-income countries scored higher in all categories of the Global Rating Scale (GRS) except the physical examination and interpretation score. Junior-grade participants scored significantly higher in history taking (junior=4.2, middle=3.7, and senior=3.7; *P*=.003) and physical examination (junior=4.0, middle=3.7, and senior=3.5; *P*=.068), but this was not significantly different. There were no statistically significant differences in GRS scores between male and female participants.

**Conclusions:**

The significant differences in clinical performance scores between low- and middle-income countries and high-income countries highlight the need for better medical education resources to bridge existing gaps in health care globally. The decrease in some clinical competency scores following career progression could be addressed by simulation-based training to maintain the same quality of history taking and physical examination skills. These outcomes, including no gendered differences in simulation-based learning, hold profound implications for tailoring medical education strategies, fostering equitable training, and elevating patient care standards on a global scale. The need for targeted interventions and capacity-building efforts via context-specific training and tailored approaches to health care education is emphasized.

## Introduction

Understanding the multifaceted global health care disparities is crucial for ensuring access to high-quality medical education [1]. The most common barriers to high-quality medical education are due to financial, resource, and accessibility inequities [2]. There is a shortage in the training resources to recruit and retain health care professionals (HCPs) in low- and middle-income countries (LMICs), leading to disparities in clinical learning opportunities compared with HCPs in high-income countries (HICs) [3]. This was further exacerbated following the COVID-19 pandemic [4,5].

E-learning and distance virtual simulation sessions can provide case-based learning and support the training of medical professionals in LMICs [6]. Specifically, initiatives using accessible technology platforms to develop high-quality educational programs can enhance postgraduate health care training [7,8]. Simulated programs provide HCPs with a learning opportunity to develop clinical competencies and improve patient outcomes in a safe and controlled environment [9,10]. However, there is an unequal distribution of simulation-based learning across LMICs, due to misconceptions regarding the cost and availability of programs [11]. Also, while the benefits of simulation training are well established, there is a paucity of research studying the differences, if any, in the clinical performance of HCPs from HICs and LMICs in simulation-based case scenarios. Understanding the similarities and differences in clinical performance can help identify areas for improvement in health care education and resource allocation in LMICs.

SIMBA (Simulation via Instant Messaging for Bedside Application) is a simulation-based learning model that uses WhatsApp and Zoom (Zoom Video Communications, Inc) platforms to improve HCPs’ confidence in managing various medical conditions [12,13]. The model is built on the principles of Kolb’s experiential learning theory and simulation gaming theory [14]. Kolb’s experiential learning theory emphasizes the importance of learning through reflection on doing, where learners actively engage in a cycle of concrete experience, reflective observation, abstract conceptualization, and active experimentation. This framework guided the design of our SIMBA sessions, ensuring that participants were exposed to real-world scenarios and encouraged to reflect on their actions and apply new knowledge in future simulations. Simulation gaming theory further shaped our curriculum by integrating gamelike elements to create an immersive and interactive learning environment. Using realistic clinical cases, role-playing, and feedback loops in our sessions aligns with the theory’s emphasis on learning through interaction and problem-solving within a simulated context. This approach fosters engagement and enhances skill development, making the learning process dynamic and participatory. It helped deliver and sustain good quality medical education during the COVID-19 pandemic [15]. It also provided alternative work experiences for premedical students [16] and a platform for medical students and junior doctors to improve teamwork and leadership skills [17]. Our previous work described how SIMBA provided equitable and high-quality postgraduate medical education to health care physicians in the LMICs and HICs [18].

As health care systems strive to provide safe and effective medical services, understanding the nuances of clinical competence among different training grades becomes a pivotal aspect of medical education and professional development. Exploring the clinical performance variations across various stages of HCP training offers valuable insights into how educational strategies and experiential learning impact the acquisition and application of clinical competencies. The need for such exploration is underscored by the potential ramifications these findings may have on patient care. An HCP’s ability to accurately diagnose, interpret diagnostic tests, make informed clinical judgments, and formulate appropriate management plans can vary across different training grades. These variations can impact patient safety, the quality of health care services, and the overall effectiveness of medical teams [19-21]. In addition, as health care systems increasingly seek to bridge global disparities in medical education and patient outcomes, it is imperative to identify how training grade–related differences influence clinical performance across diverse settings.

In this study, we explore the differences in the performance and outcomes of HCPs participating in SIMBA sessions based on gender, country of work, and training grade. This study addresses this critical knowledge gap by examining the disparities among training grades within a single context and how the geographic context of HICs and LMICs influences these disparities. The inclusion of gender as a parameter is aligned with the aspiration to tailor medical education to the diverse needs of HCPs, while also acknowledging the broader societal implications of gender-sensitive health care delivery. The study’s findings hold the potential to influence medical training practices, curriculum development, and professional mentorship in ways that are more attuned to the nuances of gender-specific experiences and perspectives.

## Methods

### Conducting SIMBA Sessions

The preparation and delivery of SIMBA sessions, including detailed flowcharts and examples, have been described in detail in our previous publication [12]. Each SIMBA session featured 4-6 clinical case scenarios representing a range of medical presentations commonly encountered in secondary care. These sessions were promoted via social media, junior doctor bulletins, and supporting organizations’ websites.

The case scenarios were adapted from real-life cases with all patient-identifiable information removed. They included comprehensive details such as presenting complaints, medical history, examination findings, clinical observations, investigation results (eg, blood tests and imaging), differential diagnoses, management strategies, and follow-up plans. Experts chairing the sessions rigorously reviewed and approved the case transcripts to ensure scientific accuracy and alignment with current medical guidelines.

Participation in the sessions was voluntary and free of charge. Participants registered in advance by providing their email addresses and WhatsApp numbers. Emails were used for presession communication, while WhatsApp facilitated real-time interaction during the simulations, providing access to all necessary session details and resources.

Before the simulation, participants completed a presimulation survey, which included informed consent, sociodemographic data collection, and self-assessment of their confidence in managing various clinical scenarios. This survey established a baseline measure of their confidence levels, covering both the session scenarios and the similar medical presentations.

During the simulation, HCPs from HICs and LMICs participated concurrently via WhatsApp. Moderators guided participants through the scenarios, encouraging them to interact as they would during real-life patient encounters. WhatsApp was also used to share key resources, such as links to surveys, investigations, and meeting invitations.

After completing all simulated cases, participants joined a Zoom meeting led by an expert. This interactive session provided an opportunity to discuss the cases, ask questions, and gain additional insights. Experts facilitated peer-to-peer discussions in the Zoom chat, fostering collaborative learning and ensuring a thorough exploration of each case.

After the simulation, participants completed a postsimulation survey, which included the confidence-rating questions from the presurvey to assess changes in confidence levels. The survey also collected feedback on their experience, highlighting successful aspects of the session and identifying areas for improvement.

WhatsApp interactions were recorded and later analyzed. Participants’ performance was objectively assessed using a GRS [22], validated by experts prior to the session. The scale evaluated 6 domains: eliciting history, physical examination, investigations and diagnostic tests, interpretation of findings, clinical judgment, and management and follow-up plans. Each domain was scored on a Likert-type scale from 1 (not done) to 5 (excellent) [23], with total scores ranging from 6 to 30. This assessment measured participants’ knowledge, accuracy, and thoroughness in handling the cases.

In addition, moderators provided personalized feedback using Pendleton’s feedback model [24,25]. Feedback highlighted participants’ strengths and offered constructive guidance for improvement, aiming to enhance their skills and confidence in managing similar scenarios in the future.

### Data Collection

**Figure 1.**
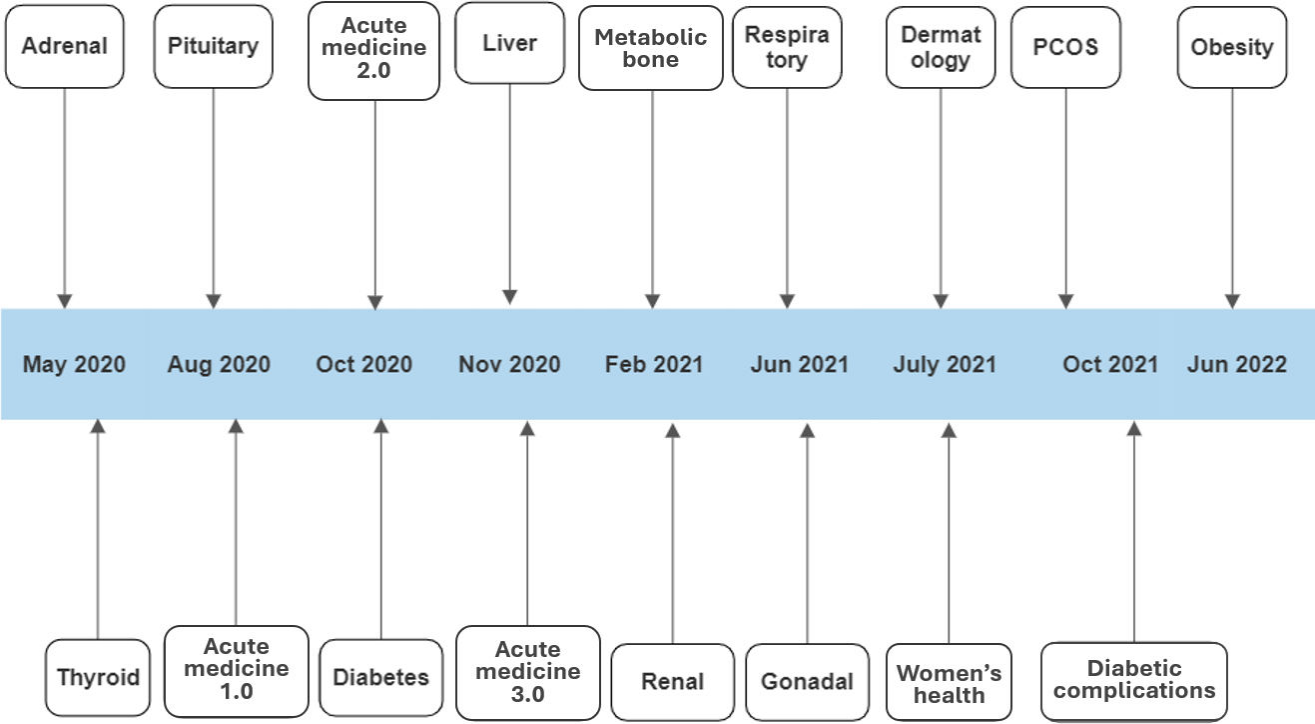
Timeline of SIMBA (Simulation via Instant Messaging for Bedside Application) sessions held from 2020 to 2022. PCOS: polycystic ovary syndrome.

This study assessed participants in 17 SIMBA sessions from May 2020 to June 2022. The topics simulated included a wide range of medical specialties, as seen in Figure 1. All participants were invited to complete both the pre-SIMBA and post-SIMBA surveys voluntarily. The pre-SIMBA survey, distributed just before the session began, gathered basic sociodemographic information and self-reported confidence levels in managing simulated cases. The post-SIMBA survey, shared immediately after the expert case discussion, included similar questions on self-reported confidence in managing these cases. It also sought participants’ feedback on their experience in the session. Each survey was administered at a single time point rather than as part of a test-retest design.

In addition, following each SIMBA session, WhatsApp chats containing the participants’ approach to the simulated scenarios were graded using an adapted version of the GRS [22], which is reviewed by experts to confirm their appropriateness for the simulated case. Our assessment tool consisted of 6 domains (eliciting history; physical examination; investigations, diagnostic tests, and imaging; interpretation of investigations and imaging; clinical judgment; and management and follow-up or discharge plan), which are rated using a Likert-type scale of 1 (not done) to 5 (excellent) [23]. Moderators calculated the participant’s score between a minimum of 6 and a maximum of 30, measuring the participant’s knowledge, accuracy, and completeness in approaching the simulated case and assessing the patient. In addition to a numerical score, moderators provided participants with written feedback based on Pendleton’s feedback model [24,25]. Pendleton’s model is a structured approach to feedback that aims to create a positive and constructive learning environment. The model emphasizes a balanced feedback process by first highlighting the learner’s strengths, then identifying areas for improvement, and finally engaging the learner in self-reflection. This structured feedback process encourages active learning and helps participants gain a deeper understanding of their performance, making it an effective tool for clinical education. GRS scores and feedback were emailed to participants 24‐48 hours after the session. In the case of any queries, participants were provided with the contact details of the SIMBA team. To ensure uniformity and fairness across scoring, all moderators attended training sessions where they were taught how to use our assessment tool by experienced moderators. Any uncertainties that arised during the scoring process were resolved by discussion with experienced moderators.

### Statistical Analysis

Participants who completed both pre-SIMBA and post-SIMBA surveys were included in the analysis. This pre-post test design with presurvey and postsurvey specifically focused on assessing changes over time rather than the consistency of responses. Participants self-reported their country of residence during surveys, and the data (countries) were grouped into HICs and LMICs according to their country of residence based on the 2022 World Bank report [26]. Participants were classified as junior, middle, and senior grades based on their work experience. Junior-, middle-, and senior-grade HCPs had 0‐2 years, 2‐5 years, and >5 years of work experience, respectively.

Unadjusted and adjusted means and 95% CIs of the scores for the 6 performance variables (history taking, physical examination, investigation, interpretation, judgment, and management) by economic classification (HIC or LMIC), level of training (junior, middle, or senior grade), and gender (male or female) were calculated using multiple linear regression models, adjusting for gender, country, training, and number of WhatsApp messages (continuous), as appropriate. The mean (95% CI) difference of the pairwise comparisons between each performance score was calculated using paired *t* tests. The *P* value for heterogeneity between the mean performance scores was calculated using likelihood ratio tests by using an ANOVA. Two-sided *P* values of <.05 were considered statistically significant. We did not adjust the *P* value for multiple comparisons. All statistical analyses were performed using the Stata statistical software package (version 27.0; StataCorp).

### Ethical Considerations

SIMBA was approved as an educational modality for postgraduate medical education by the Health Education West Midlands Diabetes and Endocrinology Specialist Training Committee. The study was approved by the Science, Technology, Engineering and Mathematics Ethics Committee at the University of Birmingham (ERN_2023‐0495 and ERN_2023‐0544). The study complied with all relevant ethical guidelines and institutional regulations throughout its design, implementation, analysis, and dissemination. Informed consent was taken from each participant and participation was voluntary. Participants were provided with an information sheet outlining the purpose of the study, the estimated duration of their involvement, the nature of the data collected, the identity of the investigators, and the storage and retention details. All data were anonymized at the point of analysis and stored on a secure, restricted access online drive. No identifiable patient data were used. No financial or material compensation was offered for participation.

## Results

### Overview

A total of 289 participants completed both pre-SIMBA and post-SIMBA surveys in the 17 simulation sessions. A total of 281 participants provided their country of residence. These participants worked in 49 countries across 5 continents, of which 36.7% (18/49) were classified as HICs, whereas 63.2% (31/49) were LMICs. Most participants worked in HICs (HICs: 191/281, 67.9%; LMICs: 90/281, 32.0%). Overall, the mean (SD) scores for the cohort were history taking: 3.6 (SD 3.9), physical examination: 3.5 (SD 3.7), investigation: 3.3 (SD 3.5), interpretation: 2.6 (SD 2.9), judgment: 3.1 (SD 3.3), and management: 2.6 (SD 2.8) (Table 1).

The values with the footnotes are the mean (95% CIs) for that particular score. Other values and corresponding *P* values are the mean difference (95% CIs) for the pairwise comparisons between scores taken from a paired *t* test for the 264 participants who had a value for all 6 outcome scores. The difference is for the performance measure on the column compared with the performance measure on the corresponding row.

**Table 1.** Mean and 95% CIs difference between various performance measures using the global rating score for those participating in Simulation via Instant Messaging for Bedside Application (SIMBA).

	History	*P* value	Physical examination	*P* value	Investigation	*P* value	Interpretation	*P* value	Judgment	*P* value	Management
History	3.7 (3.6‐3.9)^a^										
Physical examination	0.1 (0.0‐0.2)	.02	3.6 (3.5‐3.7)^a^								
Investigation	0.3 (0.2‐0.4)	<.01	0.2 (0.1‐0.3)	<.01	3.4 (3.3‐3.5)^a^						
Interpretation	1.0 (0.8‐1.2)	<.01	0.9 (0.7‐1.1)	<.01	0.7 (0.6‐0.8)	<.01	2.7 (2.6‐2.9)^a^				
Judgment	0.5 (0.4‐0.6)	<.01	0.4 (0.3‐0.5)	<.01	0.2 (0.1‐0.3)	<.01	−0.5 (−0.6 to 0.3)	<.01	3.2 (3.1‐3.3)^a^		
Management	1.0 (0.9‐1.2)	<.01	0.9 (0.8‐1.1)	<.01	0.7 (0.6‐0.8)	<.01	0.0 (−0.1 to 0.2)	.52	0.5 (0.4‐0.6)	<.01	2.7 (2.6‐2.8)^**a**^

a These values are the mean (95% CIs) for that particular score.

### Comparing the Performance Between Participants From HIC and LMIC

After adjusting for sex, training, and number of WhatsApp messages, there were statistically significant differences in performance that were identified in the categories of history taking (HIC vs LMIC: 3.8 [3.7‐3.9] vs 3.5 [3.3‐3.7]; *P*<.01), investigations (3.6 [3.5‐3.7] vs 3.1 [2.9‐3.2]; *P*<.01), clinical judgment (3.4 [3.3‐3.5] vs 2.9 [2.7‐3.1]; *P*<.01), and management (2.9 [2.7‐3.0] vs 2.3 [2.1‐2.5]; *P*<.01) (Table 2 and Multimedia Appendix 1). A slight difference, although not statistically significant, was also found in physical examination (3.7 [3.5‐3.8] vs 3.5 [3.2‐3.7]; *P*=.13) and interpretation (2.8 [2.6‐3.0] vs 2.6 [2.3‐2.9]; *P*=.23).

**Table 2.** Mean and 95% CIs of various performance measures using the global rating score for low- or middle- and high-income countries for those participating in the SIMBA (Simulation via Instant Messaging for Bedside Application).

Performance measure	Unadjusted and adjusted	Low and middle income	High income	*P* value^a^
History taking	Unadjusted	3.5 (3.2‐3.7)	3.8 (3.7‐4.0)	<.01
	Adjusted^a^	3.5 (3.3‐3.7)	3.8 (3.7‐3.9)	<.01
Physical examination	Unadjusted	3.4 (3.2‐3.6)	3.7 (3.5‐3.8)	.05
	Adjusted^a^	3.5 (3.2‐3.7)	3.7 (3.5‐3.8)	.13
Investigation	Unadjusted	3.1 (2.9‐3.2)	3.6 (3.5‐3.7)	<.01
	Adjusted^a^	3.1 (2.9‐3.2)	3.6 (3.5‐3.7)	<.01
Interpretation	Unadjusted	2.6 (2.3‐2.9)	2.8 (2.6‐2.9)	.44
	Adjusted^a^	2.6 (2.3‐2.9)	2.8 (2.6‐3.0)	.23
Judgment	Unadjusted	2.9 (2.7‐3.1)	3.4 (3.3‐3.5)	<.01
	Adjusted^a^	2.9 (2.7‐3.1)	3.4 (3.3‐3.5)	<.01

a Adjusting for country, level of training, and the number of WhatsApp messages

### Comparing the Performance Between Participants Based on Their Training Grade

A total of 213 participants provided their training grade in pre-SIMBA and post-SIMBA surveys, of which we classified 28.2% (60/213) as junior grade, 55.9% (119/213) as middle grade, and 16% (34/213) as senior grade. Junior-grade participants scored significantly higher in history taking (junior vs middle vs senior: 4.2 [4.0‐4.5] vs 3.7 [3.5‐3.9] vs 3.7 [3.4‐4.0]; *P*<.01). Otherwise, there was no significant difference across the training grade for the rest of the domains (physical examination: 4.0 [3.7‐4.2] vs 3.7 [3.5‐3.9] vs 3.5 [3.1‐3.8]; *P*=.07; investigation: 3.5 [3.3‐3.7] vs 3.4 [3.3‐3.6] vs 3.6 [3.3‐3.8]; *P*=.53; clinical interpretation: 2.6 [2.3‐2.9] vs 2.5 [2.3‐2.8] vs 3.1 [2.7‐3.6]; *P*=.08; clinical judgment: 3.4 [3.1‐3.6] vs 3.3 [3.1‐3.4] vs 3.4 [3.1‐3.7]; *P*=.63; and management: 2.8 [2.5‐3.0] vs 2.7 [2.6‐2.9] vs 2.8 [2.5‐3.1]; *P*=.94) (Table 3 and Multimedia Appendix 2).

**Table 3.** Mean and 95% CIs of various performance measures using the global rating score by level of training for those participating in SIMBA (Simulation via Instant Messaging for Bedside Application).

Performance measure	Unadjusted and adjusted	Junior grade	Middle grade	Senior grade	*P* value^a^
History taking	Unadjusted	4.2 (4.0‐4.5)	3.7 (3.6‐3.9)	3.6 (3.3‐3.9)	<.01
	Adjusted^a^	4.2 (4.0‐4.5)	3.7 (3.5‐3.9)	3.7 (3.4‐4.0)	<.01
Physical examination	Unadjusted	3.9 (3.7‐4.2)	3.7 (3.5‐3.9)	3.5 (3.1‐3.8)	.09
	Adjusted^a^	4.0 (3.7‐4.2)	3.7 (3.5‐3.9)	3.5 (3.1‐3.8)	.07
Investigation	Unadjusted	3.5 (3.3‐3.7)	3.5 (3.3‐3.6)	3.5 (3.2‐3.8)	.97
	Adjusted^a^	3.5 (3.3‐3.7)	3.4 (3.3‐3.6)	3.6 (3.3‐3.8)	.53
Interpretation	Unadjusted	2.6 (2.3‐3.0)	2.5 (2.3‐2.8)	3.1 (2.7‐3.5)	.06
	Adjusted^a^	2.6 (2.3‐2.9)	2.5 (2.3‐2.8)	3.1 (2.7‐3.6)	.08
Judgment	Unadjusted	3.3 (3.1‐3.6)	3.3 (3.2‐3.5)	3.4 (3.1‐3.7)	.96
	Adjusted^a^	3.4 (3.1‐3.6)	3.3 (3.1‐3.4)	3.4 (3.1‐3.7)	.63
Management	Unadjusted	2.7 (2.5‐3.0)	2.8 (2.6‐3.0)	2.7 (2.4‐3.0)	.87
	Adjusted^a^	2.8 (2.5‐3.0)	2.7 (2.6‐2.9)	2.8 (2.5‐3.1)	.94

a Adjusting for sex, country, and the number of WhatsApp messages.

### Comparing the Performance Between Participants Based on Their Gender

A total of 199 participants provided their gender identity in pre-SIMBA and post-SIMBA surveys (male: 83/199, 41.7%; female: 116/199, 58.3%). There were no statistically significant differences in GRS scores between male and female participants (male vs female; history taking: 3.7 [3.5‐3.9] vs 3.8 [3.6‐3.9]; *P*=.53; physical examination: 3.7 [3.5‐3.9] vs 3.6 [3.4‐3.7]; *P*=.30; investigation: 3.4 [3.3‐3.6] vs 3.4 [3.3‐3.6]; *P*=.93; clinical interpretation: 2.9 [2.7‐3.2] vs 2.7 [2.5‐2.9]; *P*=.12; clinical judgment: 3.3 [3.1‐3.5] vs 3.3 [3.1‐3.4]; *P*=.75; and management: 2.8 [2.6‐3.0] vs 2.7 [2.5‐2.8]; *P*=.42) (Table 4).

**Table 4.** Mean and 95% CIs of various performance measures using the global rating score by number of WhatsApp messages for those participating in SIMBA (Simulation via Instant Messaging for Bedside Application).

Performance measure	Unadjusted and adjusted	Men	Women	*P* value^a^
History taking	Unadjusted	3.7 (3.4‐3.9)	3.8 (3.6‐4.0)	.52
	Adjusted^a^	3.7 (3.5‐3.9)	3.8 (3.6‐3.9)	.53
Physical examination	Unadjusted	3.7 (3.5‐3.9)	3.6 (3.4‐3.8)	.35
	Adjusted^a^	3.7 (3.5‐3.9)	3.6 (3.4‐3.7)	.30
Investigation	Unadjusted	3.4 (3.3‐3.6)	3.4 (3.3‐3.6)	.89
	Adjusted^a^	3.4 (3.3‐3.6)	3.4 (3.3‐3.6)	.93
Interpretation	Unadjusted	3.0 (2.7‐3.2)	2.7 (2.4‐2.9)	.09
	Adjusted^a^	2.9 (2.7‐3.2)	2.7 (2.5‐2.9)	.12
Judgment	Unadjusted	3.3 (3.1‐3.5)	3.3 (3.1‐3.4)	.79
	Adjusted^a^	3.3 (3.1‐3.5)	3.3 (3.1‐3.4)	.75
Management	Unadjusted	2.8 (2.5‐3.0)	2.7 (2.5‐2.8)	.51
	Adjusted^a^	2.8 (2.6‐3.0)	2.7 (2.5‐2.8)	.42

a Adjusting for country, level of training, and the number of WhatsApp messages.

## Discussion

### Principal Findings

Overall, participants scored higher in history taking and physical examination skills, while their scores were lower in interpretation and management skills. This information is valuable for fine-tuning future simulation programs to focus on improving interpretation and management abilities.

GRS is a validated tool for assessing clinical competencies across 6 critical domains: eliciting history, physical examination, investigations and diagnostic tests, interpretation of findings, clinical judgment, and management and follow-up plans. Although the GRS was not used to evaluate participants’ abilities before the curriculum, it served as an objective measure during the simulation to assess performance and track growth. Combined with presimulation and postsimulation surveys, it allowed us to analyze changes in confidence levels and skill application.

The Pendleton model was adopted to provide structured feedback for the GRS output. This approach starts with positive reinforcement by highlighting what participants did well and then constructive guidance on areas for improvement. This approach ensures supportive feedback and facilitates skill enhancement.

Demographic data were collected during the presimulation survey to contextualize participants’ backgrounds and identify trends in performance or confidence changes across demographic groups. While these characteristics are static, integrating them into a pre-post framework enabled us to correlate them with dynamic variables such as confidence and skill development.

The significant differences in clinical performance scores between LMICs and HICs highlight the need for better medical education resources to bridge existing gaps in health care across the globe. A recent analysis by the General Medical Council has identified a substantial attainment gap between international medical graduates from LMIC and candidates from the United Kingdom working in the National Health Service [27]. Subsequent recommendations advise the need for tailored approaches to tackle inequality and ensure consistent quality of medical education to trainees. Free and accessible programs, such as SIMBA, can facilitate this.

The observed differences in clinical performance between HCPs from HICs and LMICs can be attributed to various factors. HCPs from HICs often benefit from better infrastructure, advanced technology, and comprehensive training programs. In contrast, LMIC professionals face limited access to resources, inadequate training opportunities, and contextual factors that may impact their clinical performance. The comparative analysis highlights the need for context-specific training and tailored approaches to health care education in LMICs. Of note, a commonly identified issue with technology-enhanced simulation is the cost of simulators and limited understanding of the use and limitations of artificial intelligence or embedded computer algorithms [28,29]. SIMBA bypasses these hurdles as it has minimal costs, and the sessions can be conducted virtually.

Whilst all training grades performed similarly across investigation, clinical judgment, and management domains, significant differences were noted in history taking and physical examination scores. The decrease in these scores following career progression suggests the need for simulation-based training for senior trainees to maintain the high quality of history taking and physical examination skills. These insights hold implications for refining medical education approaches and optimizing patient care delivery. In an era of global health care parity, comprehending training grade–related performance disparities is instrumental in fostering contextually apt and universally equitable medical training standards. A study validating the use of smartphone accelerometers in neurosurgical simulations found higher acceleration scores among junior doctors than among senior registrars and consultants, suggesting improved technical performance in junior HCPs [30]. However, our findings also contrast with findings from a study of the American Board of Internal Medicine patient satisfaction questionnaire, which found a systematic improvement in the quality of consultations in directly observed clinical skills in senior attending physicians compared with junior residents [31]. The decline in history and examination skills may stem from increased workload, encouraging reliance on diagnostic investigations over thorough assessments. Deskilling—reduced use of certain skills in practice—also contributes to a decline in history and examination skills. The potential reason for contrast with findings from various national and international educational boards likely arises from differences in settings; formal assessments occur in controlled environments, encouraging conscious performance. In contrast, SIMBA sessions reflect real-life scenarios without direct observation, emphasizing authentic application of skills, which may reveal different trends.

Recommendations by Health Education England highlight the growing role of simulation-based education in core medical training, including procedural and emergency presentation learning sessions [32]. Its widespread use and implementation of a clear framework across all National Health Service trusts may allow physicians to further develop their skills and knowledge in line with up-to-date guidelines throughout their careers.

We did not find a significant difference in GRS between male and female participants. This is consistent with previous findings, which showed no significant gender-related differences in simulation assessment scores among emergency medicine residents assessed against 2 simulation cases related to the emergency medicine curriculum [33]. Similarly, simulation-based learning has been shown to eliminate gender differences in a virtual surgical training course in endoscopic proficiency [34]. However, while gender differences in the simulation were investigated, the translation of simulated skill to physical performance in the endoscopy suite was not investigated. Consequently, further prospective studies are required to assess the impact of gender on clinical performance. Previous studies have further demonstrated the role of simulation-based education in developing interpersonal communication skills and teamwork among medical students and HCPs [35,36]. Therefore, simulation-based learning may provide objective and standardized scenarios to help remove gender-based differences in clinical outcome scores.

While mobile simulation-based learning appears to be widely accessible, reliable web access and cost of access continue to pose a barrier to virtual learning in many LMICs [37]. This is a particular issue in remote areas of the world, which may be subject to unscheduled power cuts [38]. Although communication platforms such as WhatsApp serve as low-cost initiatives for delivering medical education, emphasis must be placed on data security and confidentiality [39]. Consequently, standardized guidelines concerning the use of instant messaging in health care education are required to ensure its sustainability [40].

### Limitations

We did not assess the change in perception toward virtual education over time. This may have influenced the findings of experience and difficulties that the participants encountered with web-based education from both LMICs and HICs. Future longitudinal studies must determine whether subsequent clinical decision-making is improved in real-life scenarios. Brain drain, that is, migration of doctors usually from LMICs to HICs for better opportunities and other varied reasons, is another important factor that needs to be considered as this phenomenon may have skewed the results. As SIMBA sessions are currently conducted only in English, it is a potential language barrier. As all the data collected were self-reported rather than objectively verified or tested, it is an inherent limitation of the study.

Regarding the physical examination, participants requested specific types of examinations, such as general physical examination, abdominal examination, or limb examination, and the corresponding findings were provided to them. The virtual clinical physical examination was not performed by the participants themselves, which is a limitation of our study. As we collected minimal identifying data, we do not have further information on the career breaks taken by a doctor for any reason that may have led to inaccurate classification as a junior, middle, or senior-grade doctor. Moreover, given the growing role of virtual learning in medical education, future work should focus on user perception of the feasibility of remote simulation-based training in LMICs.

### Conclusions

Future simulation programs should enhance interpretation and management skills by incorporating customized content, feedback mechanisms, and real-world scenarios for HCPs. The significant differences in clinical performance scores between LMICs and HICs highlight the need for better medical education resources to bridge existing gaps in health care globally. The decrease in some clinical competency scores following career progression could be addressed by simulation-based training to maintain the same quality of history taking and physical examination skills. These outcomes, including no gendered differences in simulation-based learning, hold profound implications for tailoring medical education strategies, fostering equitable training, and elevating patient care standards on a global scale. The need for targeted interventions and capacity-building efforts via context-specific training and tailored approaches to health care education is emphasized.

## Supplementary material

10.2196/52332 Multimedia Appendix 1 Global Rating Scale score of HIC versus LMIC participants (statistically significant results in history taking, investigations, clinical judgment, and management). HIC: high-income country; LMIC: low- and middle-income country.

10.2196/52332 Multimedia Appendix 2 Global Rating Scale score of participants by training grade (statistically significant results in history taking).

## Abbreviations

HCPs: health care professionals
HICs: high-income countries
LMICs: low- and middle-income countries
SIMBA: Simulation via Instant Messaging for Bedside Application
