# Supplementary figures and images for: Global Disparities in Simulation-Based Learning Performance: Serial Cross-Sectional Mixed Methods Study
Source: JMIR Med Educ. 2025 Aug 11;11:e52332. doi: 10.2196/52332 (PMC12338850; doi:10.2196/52332)

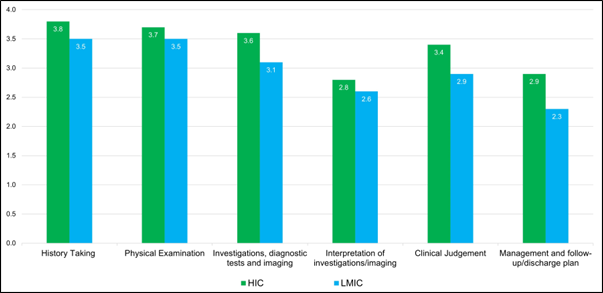

Supplement: Multimedia Appendix 1 [file mededu-v11-e52332-s001.png]

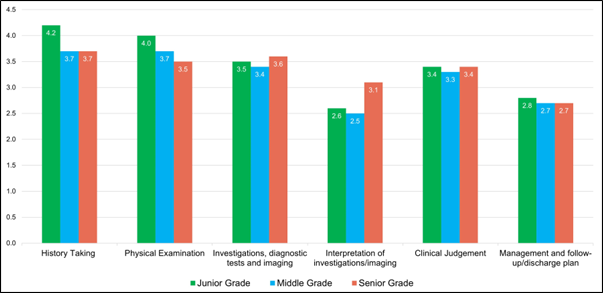

Supplement: Multimedia Appendix 2 [file mededu-v11-e52332-s002.png]
